# A Worldwide Bibliometric Analysis of Tetrandrine Research in Recent Two Decades

**DOI:** 10.3389/fphar.2022.896050

**Published:** 2022-06-15

**Authors:** Guang Yang, Wenqing Xie, Yilan Ding, Weiyang Wang, Cheng Huang, Tingxiao Zhao, Yusheng Li

**Affiliations:** ^1^ Department of Orthopedics, Xiangya Hospital, Central South University, Changsha, China; ^2^ Xiangya School of Medicine, Central South University, Changsha, China; ^3^ Department of Orthopedics, China-Japan Friendship Hospital, Beijing, China; ^4^ Center for Plastic and Reconstructive Surgery, Department of Orthopedics, Zhejiang Provincial People’s Hospital (Affiliated People’s Hospital, Hangzhou Medical College), Hangzhou, China; ^5^ National Clinical Research Center for Geriatric Disorders, Xiangya Hospital, Central South University, Changsha, China

**Keywords:** bibliometric, tetrandrine, cancer, pharmacokinetics, arthritis, VOSviewer, RStudio

## Abstract

**Background:** Tetrandrine has been the focus of many studies in recent years. Currently, no bibliometric study in this field has been published. This study presents a bibliometric analysis of the articles on tetrandrine research from the WOS core database during the recent two decades.

**Methods:** Documents were retrieved for further bibliometric analysis based on the search terms: [TI = (Tetrandrine OR Sinomeninea OR Hanfangchin A) AND PY = (2000–2021)]. We used Microsoft Excel to conduct the frequency analysis, VOSviewer for data visualization, and RStudio for citation metrics and analysis. The standard bibliometric indicators such as the temporal trends and geographical distribution of publications and citations, prolific authors and co-authorship, keywords citation burst, preferred journals, top-cited articles, and important institutions were applied in this study.

**Results:** 490 documents were retrieved from WOS core database, the retrieved document type consists of 8 categories: 425 articles, 42 meeting abstracts, 8 reviews, 7 corrections, 3 editorial material, 2 proceedings paper, 1 letter, 1 retraction. Corrections and Retractions was excluded from this investigation, the left 482 document were included for furter bibliometric analysis.

**Conclusion:** Based on our findings, there was a continuous growth of publications on tetrandrine research for 22 years since 2000. China was the largest contributor to tetrandrine research, followed by the United States. The most influential author was Cheng Y (Natl Taiwan Univ Hosp). Acta Pharmacol Sin remained the main publication related to tetrandrine research. Chinese Academy of Sciences, is expected to be a good collaborating center in tetrandrine research. The use of tetrandrine in cancer treatment, could be the promising research subject areas to follow.

## 1 Introduction

Tetrandrine (TET) is a bisbenzylisoquinoline alkaloid originally purified by Kondo and Yano from a medicinal plant, Stephania tetrandra S. Moore (Menispermaceae), and validated by the following researchers Chen and Chen (1935). Stephania tetrandra S. Moore plants are herbaceous or woody vines. Approximately 60 species have been identified in tropical and subtropical Asia and Africa, with a few species being found in Oceania. In China, 37 species (30 of which are indigenous) have been recognized (Hu et al., 1996). The growth cycle of Stephania plants are more than 3 years. Weak in self-recovery ability after mining, the Stephania tetrandra used for medical purposes depend entirely on the supply of wild resources (Jiang et al., 2020).

Stephania tetrandra S. Moore has been incorporated into the Chinese Pharmacopoeia, known as “Fangji.” Fangji was first mentioned in the Shennong Bencao Jing, a classic compendium of traditional Chinese medicine created during the Qin and Han Dynasties (100 BC-200 AD) (Zhang et al., 2020). So far, several prescriptions contain Fangji, which has a significant therapeutic role in China patent medications such as Shi-Wei-Feng-Xiao Capsules (Wei and Zhao, 2013) and Qi-Fang-Xi-Bi-Granules (Xingang et al., 2015). Several medicinal properties compounds have been isolated from Fangji, including alkaloids, flavonoids, and steroids, alkaloids are the principal active components in Fangji. Among all alkaloids isolated from Fangji, TET is a compound with broad pharmacological activity and potential research prospects (Cai et al., 2011).

TET, a colorless rod-shaped crystal, is a bisbenzylisoquinoline alkaloid, insoluble in water, benzene reagents and dissolve easily in chloroform, methyl alcohol. The molecular formula and molecular weight of TET are C_38_H_42_O_6_N_2_ and 622.3 (Chen and Tseng, 2010). Methods for extracting bisbenzylisoquinoline alkaloid from the roots of Stephania tetrandra including Column Chromatography (Kupchan and Altland, 1973) and Ionic Liquid Based Ultrasound Assisted Extraction method (Zhang et al., 2015). However, most procedures regarding extraction and purification of TET from Stephania tetrandra root are basically adaptations of Chen’s approach (Chen and Chen, 1935).

TET is normally used in traditional Chinese medicine prescription as an analgesic and diuretic agent and widely applied in the treatment of rheumatism, arthralgia, edema, and beriberi, unfavorable urination, and eczema (Jiang et al., 2020). With the further study, the medicinal activities in hepatic cells protecting, hepatic fibrosis resistance, portal hypertension reduction, tumor cells apoptosis induction and multidrug resistance reversal entered researchers’ vision (Cai et al., 2011). In recently years, researchers have proved its anticancer properties both *in vitro* and *in vivo*, including colorectal cancer (He et al., 2011), endometrial cancer (Shang et al., 2021), breast cancer (Guo et al., 2020), pancreatic cancer (Singh et al., 2016), bladder cancer (Zhang et al., 2016) and laryngeal cancer (Cui et al., 2019), cancer angiogenesis and metastasis (8), in addition, TET’s effects in cancer angiogenesis and metastasis suppression has also been reported (Gao et al., 2013).

Since the first publication of TET, there has been a surging number of TET related studies, study topics such as the extraction method of tetrandrine, anti-tumor mechanism of TET occupied the majority. TET research has presented diversified characteristics, however, the lack of literature on systematic analysis of the research status makes the investigation in this field operated without a compass. Currently, no studies have analysis the publication trend and research hotspots of TET and bibliometrics can change that situation. Bibliometrics refers to the interdisciplinary science of quantitative analysis of all knowledge carried by means of mathematics and statistics. Through the statistical analysis of relevant literature, researchers can obtain the nature and development direction of a certain discipline. This article aims to provide a view of global trends in tetrandrine research *via* the bibliometric analysis through WOS database. We explored standard bibliometric indicators such as the temporal trends and geographical distribution of publications and citations, prolific authors and co-authorship, keywords citation burst, preferred journals, top-cited articles and important institutions.

## 2 Materials and Methods

### 2.1 Data Source and Search Strategy

**FIGURE 1:**
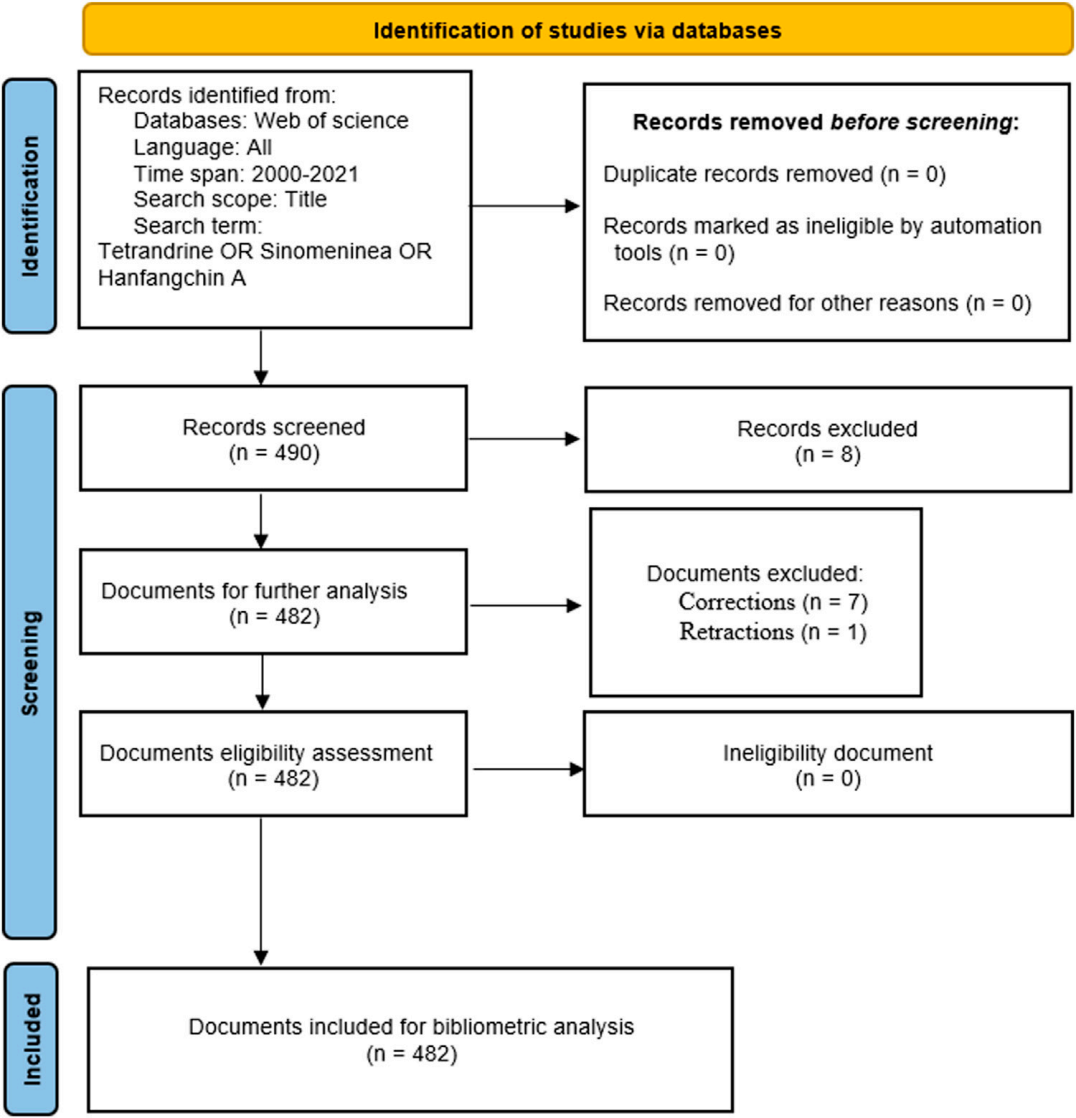
Flow diagram of the search strategy.

Bibliometric analysis in tetrandrine was performed using the WOS core database as of December 2021. The search terms, [TI = (Tetrandrine OR Sinomeninea OR Hanfangchin A) AND PY = (2000–2021)], were used to search for relevant articles published in any language related to tetrandrine research. We focus on the title of the article to ensure a preferable search result that juggle accuracy and comprehensiveness. As the component of the article, the title summarizes other sections of the article and becomes the face of the article. It represents the article’s main content that is convenient for us to identify relevant article from the WOS database. We refined the time range to publishing year from 2000 to 2021 to analyze the recent trend in tetrandrine research field. Two researchers searched according to the search terms *via* WOS database, and the third researcher was invited to participate the searching work when the search results were diversified. Figure 1 shows our search strategy.

### 2.2 Information Extraction

To avoid double error counting and negative results two different researchers were assigned to complete a solo retrieval task. All the retrieved documents were used for bibliometric analysis. Four different kinds of software were used in this study:

1) Microsoft Excel 2019 for calculating the frequencies and percentage of the published materials.
2) VOSviewer (version 1.6.17) for the bibliometric networks.
3) RStudio to calculate the citations metrics.
4) CiteSpace for keywords citation burst analysis.

### 2.3 Relevant Bibliometric Indicators

H-index: The H-index reflects H papers published by the journal/author/country, each of which has been referenced at least H times. It can be applied in evaluating the scientific influence of the journal, author, or country.

G-index: Researcher gained G-index = G means there were G articles have been cited at least G^2^ times.

$$M-\mathrm{index}=\frac{H-\mathrm{index}}{Y_{\mathrm{academic}\;\mathrm{age}}}$$

DF, Dominance Factor, the frequency with which the author is the first author of a co-authored article.

## 3 Results

### 3.1 Description of Retrieved Literature

A total of 490 articles were identified from the WOS database and 482 articles consistent with the inclusion criteria. Articles type such as Correction and Retraction were excluded from the study. The retrieved document type consists of 8 categories: review article, meeting abstract, correction, editorial material, proceedings paper, letter, retraction. Table 1 summarizes the constitution of the repertoire depending on the type of document. In all the documents, the original documents (425, 86.73%) dominated the list, followed by meeting abstracts (42, 8.57%). Review articles (9, 1.84%), Corrections (7, 1.43%) contribute more than 1% of the total publications, and other document types that each only contributed less than 1% of the total publications. Corrections and Retractions was excluded from this investigation, therefore, 482 documents were available for this study.

**TABLE 1:** Types of retrieved documents (2000–2021).

Document type	Total publications (TP)	Percentage (%)
Article	425	86.73
Meeting abstract	42	8.57
Review	9	1.84
Correction	7	1.43
Editorial material	3	0.61
Proceedings paper	2	0.41
Letter	1	0.20
Retraction	1	0.20
Total	490	100.00

### 3.2 Temporal Trends of Publications and Citations

**FIGURE 2:**
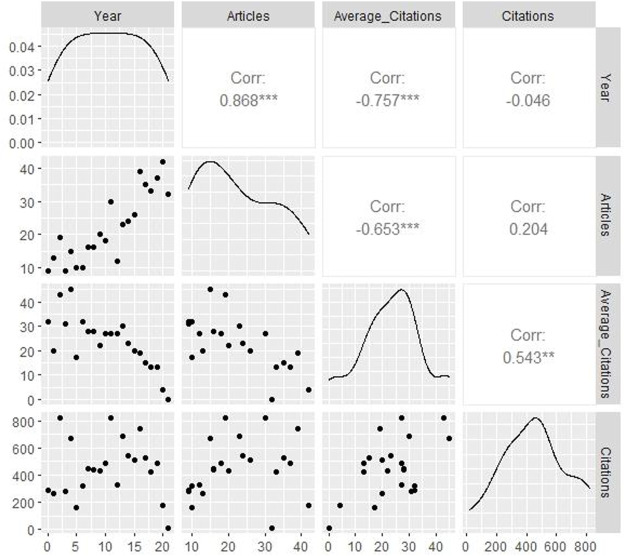
Matrix scatter diagram.

Relevant data of annual scientific production in TET research field were represented in Table 2, the highest productivity was observed in 2020, with a total of 42 documents, and the lowest productivity was in 2000 and 2003, with a total of 9 documents. The highest publication growth rate during the investigated period located in 2015–2016, with 50%. There was a significant decline in articles productivity from 2011 to 2012, articles produced in 2012 was about over 50 percent lower than in 2011. Despite the annual article publications in the investigated period showed a fluctuant trend, an overall increasing trend in publications can also be observed. The citation matrix per year for retrieved documents is shown in Table 2. The highest total citation was observed in 2002, with a total of 820 citations, followed by 2011 with a total of 819 citations. Articles published in latest years such as 2021 and 2020 gained lower total citations, with respectively a total of 15 and 180 citations. During the investigated period, articles published in 2004 were seemingly more popular, with average 44.73 citations per article. Articles published in the latest 5 years gained few tractions from researchers, the average citations per article is less than 20 times, among them, 2021 was the lowest, with 0.47 citations per article. In Figure 2, “Average Citations” were negatively correlated with “Years,” the overall positive correlation between “Years” and “Articles” can also be observed, there was no significant correlation between other indicators.

**TABLE 2:** Annual scientific productions and citations.

Year	Actual value			Normalized value		
	Articles	Average citation	Citations	Articles	Average citations	Citations
2000	9	32	287	0.00	0.71	0.34
2001	13	20	263	0.12	0.45	0.31
2002	19	43	820	0.30	0.96	1.00
2003	9	31	283	0.00	0.70	0.33
2004	15	45	671	0.18	1.00	0.81
2005	10	17	165	0.03	0.36	0.19
2006	10	32	319	0.03	0.71	0.38
2007	16	28	452	0.21	0.63	0.54
2008	16	28	443	0.21	0.61	0.53
2009	20	22	432	0.33	0.48	0.52
2010	18	27	492	0.27	0.61	0.59
2011	30	27	819	0.64	0.61	1.00
2012	12	27	326	0.09	0.60	0.39
2013	23	30	688	0.42	0.67	0.84
2014	24	23	542	0.45	0.50	0.65
2015	26	20	512	0.52	0.43	0.62
2016	39	19	741	0.91	0.42	0.90
2017	35	15	526	0.79	0.33	0.63
2018	33	13	426	0.73	0.28	0.51
2019	37	13	492	0.85	0.29	0.59
2020	42	4	180	1.00	0.09	0.20
2021	32	0	15	0.70	0.00	0.00

### 3.3 Prolific Authors and Co-Authorship

**FIGURE 3:**
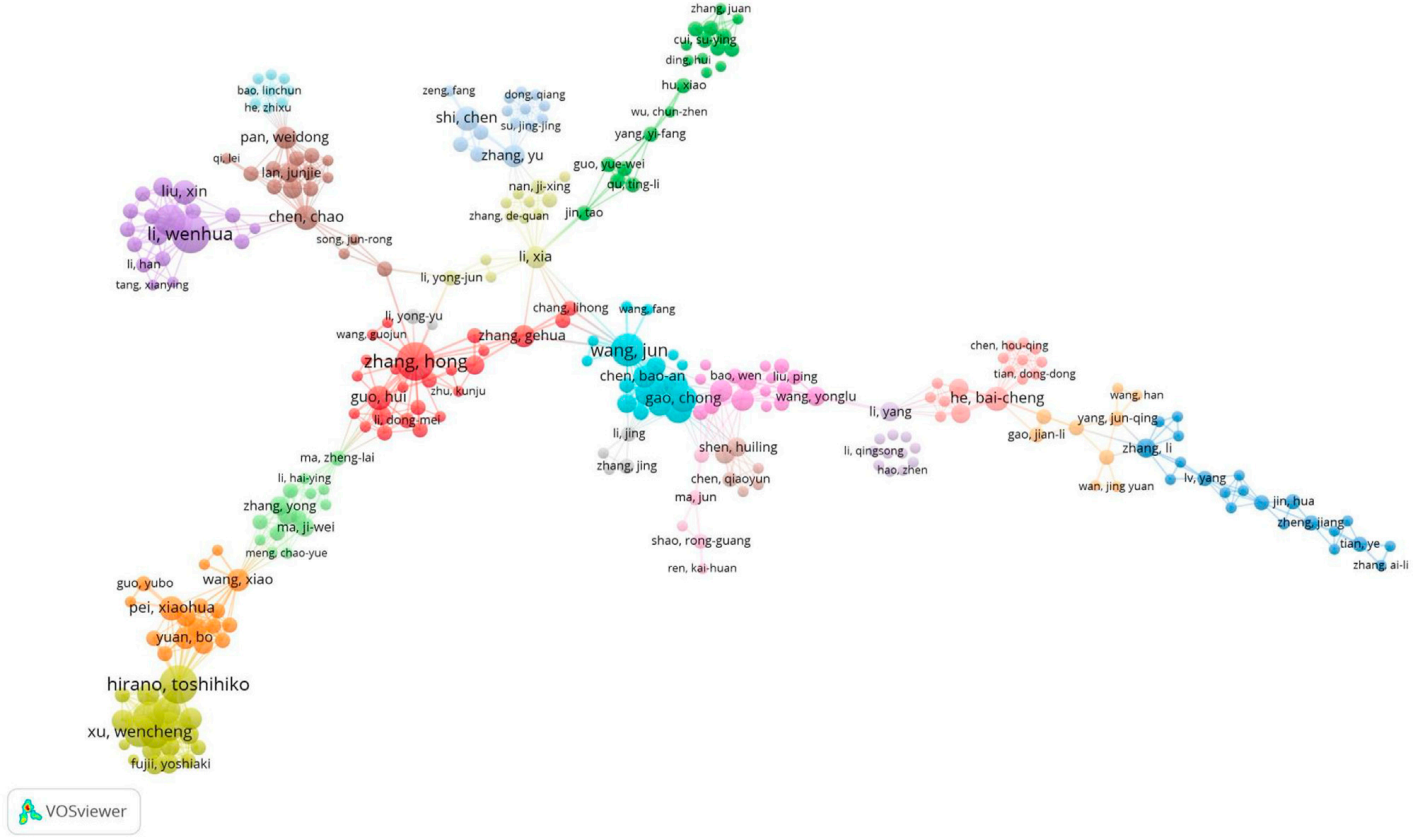
Network visualization map of co-authorship in TET research.

A total of 1885 authors contributed to TET research. 9 documents were single-authored publications while the remaining documents were multi-authored publications. The maximal author count of a single article is 24, most articles were accomplished by 5–8 authors. Therefore, the prevalence of team collaboration or the degree of research cooperation among TET researchers was 98%. Table 3 lists the top 10 authors distributed by publications and citations during the investigated period. Zhang H. (Jinan University, Affiliated Hosp 1) with a total of 19 articles was the most productive author, followed by Hirano T. (Tokyo University) with a total of 14 documents. Cheng Y. (Natl Taiwan University Hosp) was the most productive first-author with a total of 8 dominant articles (DF = 0.73). Chen BA won the Rank 2 (DF Rank) with a total of 6 first author articles (DF = 0.67). The top three most cited authors were Li WH (547), Liu X (396), Liu T (380). Li WH (Wuhan University) is the most cited authors in TET research with a total of 547 citations. Li WH’s H, G and M index was respectively 11, 11, 0.92. Wuhan University and Sun Yat Sen University were the popular institution in TET research field. Authors with minimum productivity of 5 documents and a minimum total citation of 10 were visualized using the VOSviewer technique (Figure 3). Research teams such as Zhang H’s team, Wang J’s team, Li WH’s team, and Hirano T’s team made significant contribution to TET research.

**TABLE 3:** Top 10 authors distributed by publications and citations.

Rank	Author	Publications	Country	Institution	First author	DF	DF rank	Author	Country	Institution	H-Index	G-Index	M-index	TC	Publications	PY_start
1	Zhang,H	19	China	Jinan Univ, Affiliated Hosp 1	4	0.21	7	Li WH	China	Wuhan Univ	11	11	0.92	547	12	2011
2	Hirano,T	14	Japan	Tokyo Univ	5	0.36	4	Liu X	China	Wuhan Univ	7	8	0.58	396	8	2011
3	Wang,J	14	China	Southeast Univ, ZhoDa Hosp	3	0.21	5	Liu T	China	Wuhan Univ	9	9	0.90	380	9	2013
4	Zhang,Y	14	China	Jinan Univ	3	0.21	6	Chen Y	China	Natl Taiwan Univ Hosp	8	10	0.57	353	11	2009
5	Koul,S	13	America	Univ Colorado	7	0.54	3	Fu LW	China	Sun Yat Sen Univ	3	3	0.14	322	3	2002
6	Li,WH	12	China	Wuhan Univ	0	0.00	9	Liang YJ	China	Sun Yat Sen Univ	3	3	0.14	322	3	2002
7	Chen,Y	11	China	Natl Taiwan Univ Hosp	8	0.73	1	Pan QC	China	Sun Yat Sen Univ	2	2	0.10	272	2	2002
8	Chen,J	10	China	Huazhong Univ	2	0.20	8	Yang XP	China	Sun Yat Sen Univ	2	2	0.10	272	2	2002
9	Chen,BA	9	China	Southeast Univ	6	0.67	2	Gong K	China	Wuhan Univ	3	3	0.25	266	3	2011
10	Koul,HK	9	America	Univ Colorado	0	0.00	10	Tseng SH	China	Taiwan Univ Hosp	8	9	0.57	259	9	2009

PY_Start: the year that researcher published his first article during the investigated period.

### 3.4 Geographical Distribution of Publications and Citations

**FIGURE 4:**
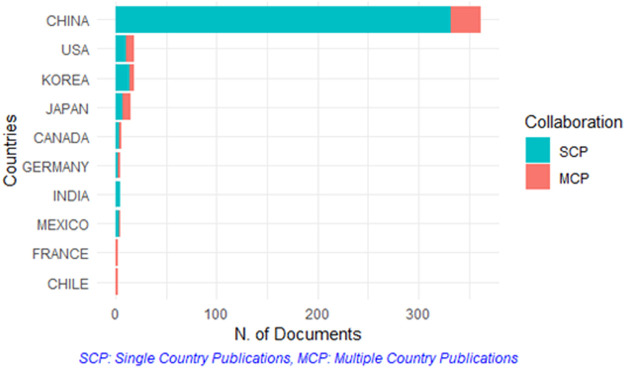
Publication distributed by single country publications and multiple country publications.

**FIGURE 5:**
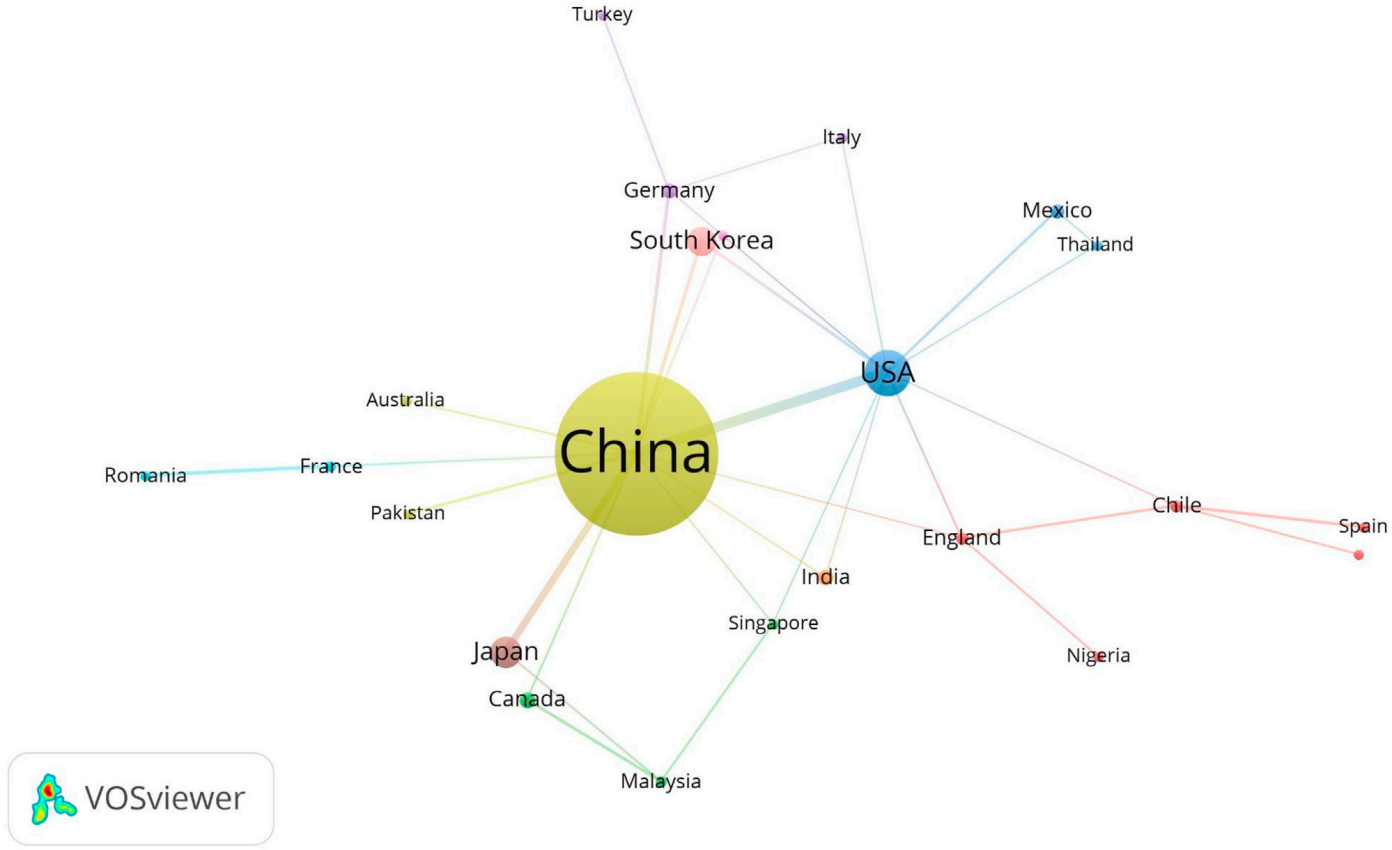
Network visualization map of international collaboration among countries. The thickness of the connecting line between any two countries indicates the strength of collaboration. Countries with similar colors form one cluster.

Researchers from 23 different countries contributed to the publication of retrieved documents. The top 10 countries contributed to article publications were represented in Table 4. China gained Rank 1 with a total of 362 articles (332 articles was single country publication) followed by the United States with a total of 19 articles (11 articles was single country publication). The bottom five countries have fewer than 10 articles showed no significance to our analysis. Figure 4 represents the articles distribution based on single country publications and multiple country publications. Articles from some countries such as Canada, Germany, India, Mexico, France, and Chile were basically composed of multiple country cooperative publications. With a total of 6,398 citations, China was the most cited country, Korea with a total of 577 citations gained the Rank 2 in the most cited list. However, the number of articles published in left countries stayed at a low volume and reflected no statistically significance. The countries cooperation network is shown in Figure 5. China, as an important contributing member in TET research field, has relatively frequent cooperation with the United States and Japan. Although articles from South Korea were ranked third in total Citations, cooperation between South Korea and other countries was not frequent.

**TABLE 4:** Top 10 Country distributed by publications and citations.

Rank by publications	Country	Articles	Citations	Average citations (AC)	Percentage (%)	SCP	MCP	Rank by AC
1	China	362	6398	17.67	80.09	332	30	6
2	United States	19	473	24.89	4.20	11	8	4
3	South Korea	18	577	32.06	3.98	14	4	1
4	Japan	15	174	11.6	3.32	7	8	7
5	Canada	6	120	20	1.33	4	2	5
6	Germany	5	135	27	1.11	2	3	3
7	India	5	155	31	1.11	5	0	2
8	Mexico	5	38	7.6	1.11	4	1	8
9	France	3	0	0	0.66	0	3	9
10	Chile	2	0	0	0.44	0	2	10

SCP, single country publications; MCP, multiple country publications.

### 3.5 Analysis of Keywords

#### 3.5.1 Hotspot Summarized From Keywords

Rstudio was used to calculate keywords from 482 retrieved documents, the total number of Author Keywords (DE) and Author Keywords-Plus (ID) is 1,082 and 1,363, respectively. In Author Keywords (DE) group, the keywords used at high frequency were: “Tetrandrine,” “Apoptosis,” “Autophagy,” “Fangchinoline,” “NF-Kappa B,” “Multidrug Resistance,” “P-Glycoprotein,” “Pharmacokinetics,” “Proliferation,” “Tetrandrine (Tet).” Keywords used at high frequency in Author Keywords-Plus (ID) group were: “Apoptosis,” “*In-Vitro,*” “Expression,” “Activation,” “Cells,” “Multidrug-Resistance,” “Inhibition,” “Mechanisms,” “Cancer,” “Growth”. The frequency results are presented in Table 5.

**TABLE 5:** Keywords distributed by frequency.

Rank	Author keywords (DE)	Frequency	Keywords-plus (ID)	Frequency
1	Tetrandrine	326	Apoptosis	86
2	Apoptosis	68	*In-Vitro*	68
3	Autophagy	21	Expression	67
4	Fangchinoline	17	Activation	59
5	NF-Kappa B	16	Cells	50
6	Multidrug resistance	15	Multidrug-Resistance	50
7	P-Glycoprotein	14	Inhibition	46
8	Pharmacokinetics	14	Mechanisms	41
9	Proliferation	13	Cancer	35
10	Tetrandrine (TET)	10	Growth	34

#### 3.5.2 Subdisciplines Classified by Keywords

**FIGURE 6:**
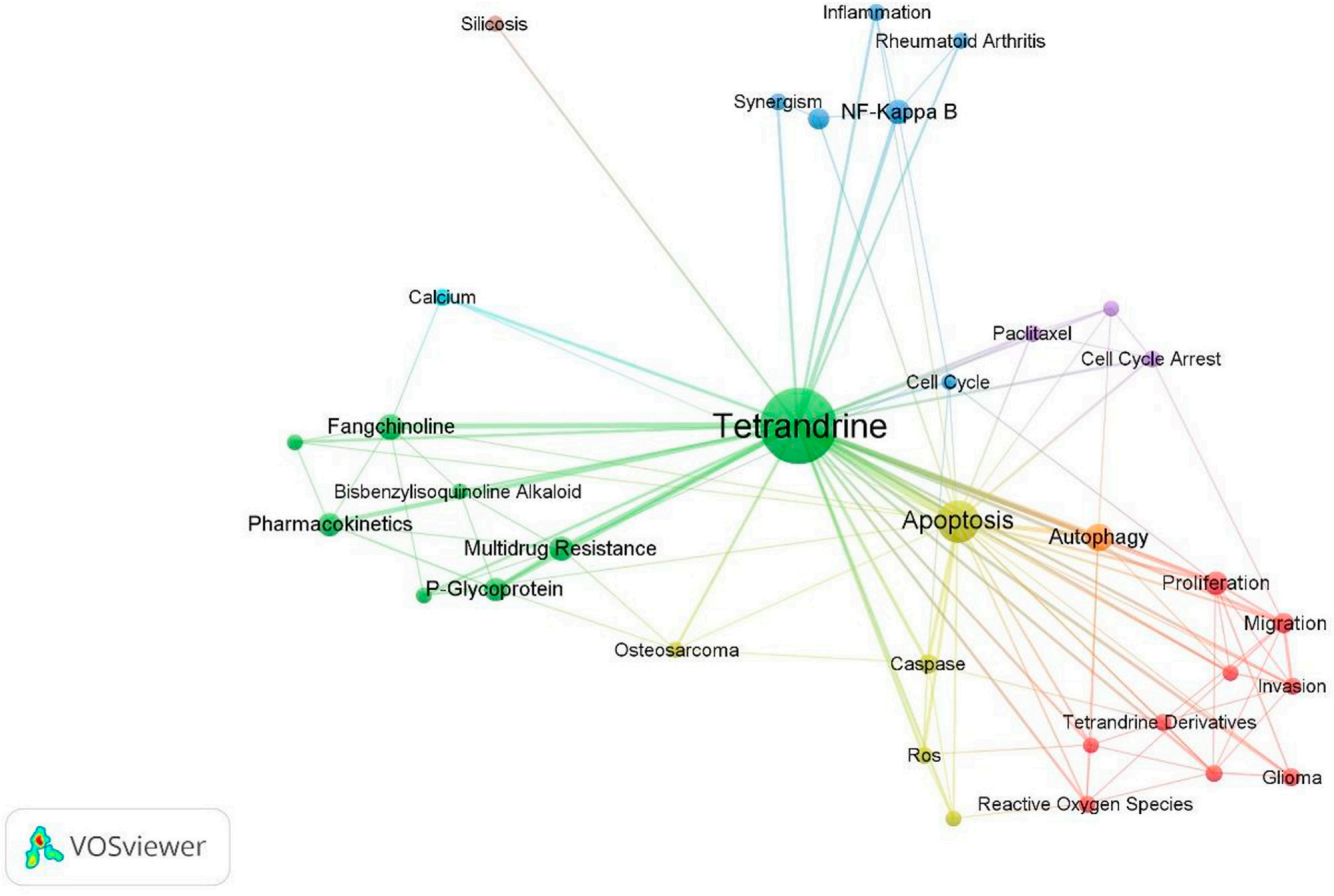
Network visualization map of the author keywords. keywords with similar colors form one cluster.

**FIGURE 7:**
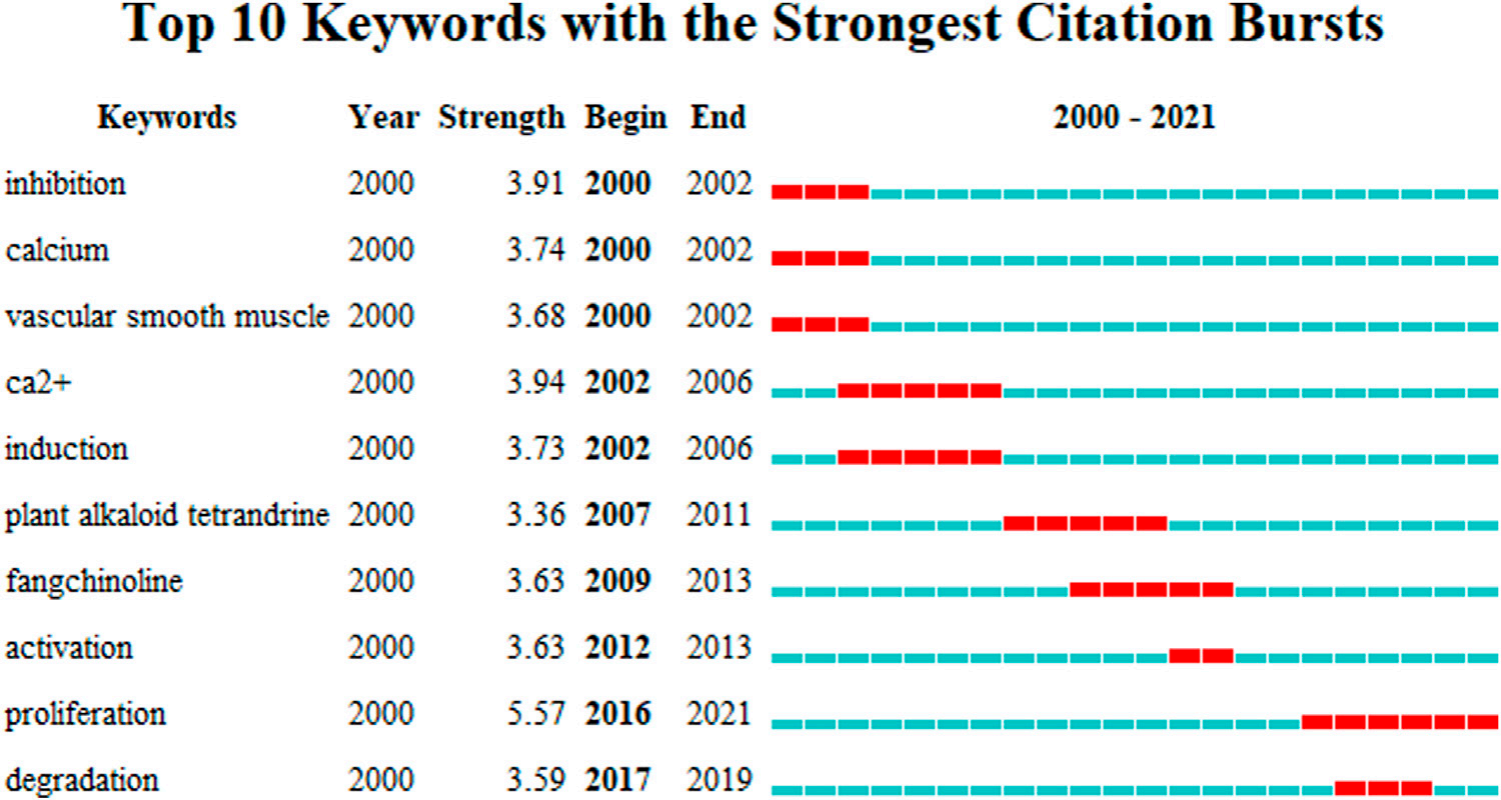
Top 10 keywords with the strongest citation bursts.

Mapping with the VOSviewer technique of author keywords with minimum occurrences of 10 showed that ones such as “Tetrandrine,” “Apoptosis,” “Autophagy” were the top 3 occurrences author keywords (Figure 6). Keywords that met the screening criteria were divided into eight clusters, Circles in the same color cluster suggest a similar topic among the publications. Each represents a subfield of the field of tetrandrine research. Specifically, as was shown in the red cluster (cluster 1,9), keywords such as “Akt,” “Angiogenesis,” “Glioma,” “Invasion,” “Migration,” “Proliferation,” “Prostate Cancer,” “Reactive Oxygen Species,” “Tetrandrine Derivatives apoptosis” are related to the topic “oncology.” Keywords in the green cluster (cluster 2,8) such as “Bisbenzylisoquinoline Alkaloid,” “Cytotoxicity,” “Fangchinoline,” “Multidrug Resistance,” “P-Glycoprotein,” “Pharmacokinetics,” “Stephania Tetrandra,” “Tetrandrine” are related to the topic “pharmacology.” Keywords in the blue cluster (cluster 3,6) include “Cell Cycle,” “Inflammation,” “NF-Kappa B,” “Rheumatoid Arthritis,” “Synergism,” “Tetrandrine” are related to the topic “arthritis.” Keywords in other clusters provide insufficient information and contribute to obstacle in summarizing the corresponding subdiscipline classification. Figure 7 depicts the top 10 keywords with the strongest citation bursts, keyword “proliferation” was the most cited keyword in recent years.

### 3.6 Preferred Journals

**FIGURE 8:**
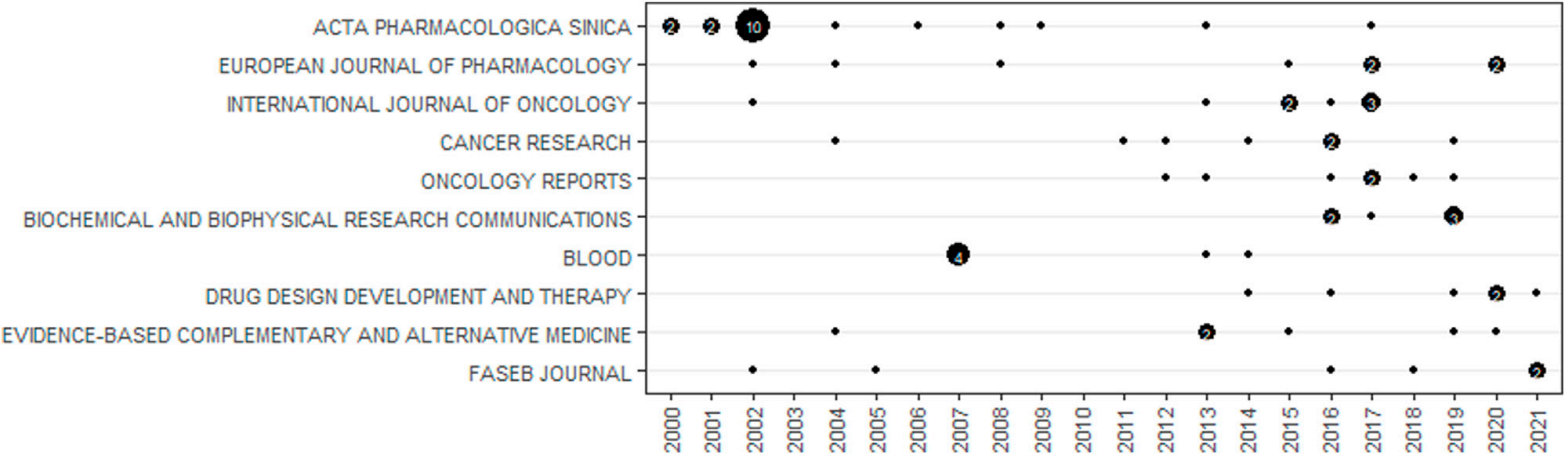
Visualization of Top 10 prolific journals publications distribution according to the temporal order. Publications with less than 2 are presented in the form of black dot. Publications with more than 2 are represented in number form.

Table 6 lists the top 10 journals distributed by publications and total citations on TET research. With a total of 21 articles, Acta Pharmacol Sin was the most productive journal. The second most productive journal was Eur J. Pharmacol with a total of 8 articles, followed by Int J. Oncol with a total of 8 articles. The remaining journals on the list all only contained 6–7 articles. According to the 2020 JCR report, Acta Pharmacol Sin, Cancer Res and Blood are all included in Q1. Blood, though it gained IF = 23.63 and was included in Q1, did not dominate the TET research field. Acta Pharmacol Sin was also the most cited journal in TET research field with a total of 651 citations and 14 H-index, additionally, Acta Pharmacol Sin was also the journal with the lowest average citation. Cancer Chemoth Pharm was the second most cited journal with only 4 articles, followed by Int J. Oncol with 272 citations and 8 articles. Figure 8 visualizes the journal publications in chronological order. During the first half of the investigated period, Acta Pharmacol Sin was the dominant journal in TET research field, between 2015 and 2021, it faded from the leading journal.

**TABLE 6:** Top 10 journals distributed by publications and citations.

Rank	Journal	Publications	% Of 482	IF(JCR 2020)	JIF quartile	Journal	Total citations	Publications	Average citations (AC)	H-Index	IF(JCR 2020)	JIF quartile	AC rank
1	Acta pharmacol sin	21	4.36	6.15	Q1	Acta pharmacol sin	651	21	31	14	6.15	Q1	10
2	Eur J. pharmacol	8	1.66	4.43	Q2	Cancer chemoth pharm	280	4	70	4	3.33	Q3	2
3	Int J. oncol	8	1.66	5.65	Q2	Int J. oncol	272	8	34	8	5.65	Q2	9
4	Cancer res	7	1.45	12.70	Q1	Biochem pharmacol	234	5	47	5	12.70	Q1	7
5	Oncol rep	7	1.45	3.91	Q3	Mol pharmaceut	234	4	59	4	3.91	Q3	4
6	Biochem bioph res Co.	6	1.24	3.58	Q3	Mol pharmacol	227	3	76	3	3.58	Q3	1
7	Blood	6	1.24	23.63	Q1	Cancer lett	190	4	48	4	8.68	Q1	5
8	Drug des dev ther	6	1.24	4.16	Q2	J. ethnopharmacol	188	4	47	4	4.36	Q2	6
9	Evid-based compl alt	6	1.24	2.63	Q2	Int J. cancer	183	3	61	2	7.40	Q1	3
10	Faseb J.	6	1.24	5.19	Q2	Life sci	181	4	45	4	5.04	Q2	8

### 3.7 Analyze of Highly Cited Articles

The top 10 cited articles in TET research field are shown in Table 7, the top three cited articles were as follows. 1) “The multidrug resistance of tumor cells was reversed by tetrandrine *in vitro* and in xenografts derived from humanbreast adenocarcinorna MCF-7/adr cells”: multidrug resistance is one of the most significant roadblocks to the efficacy of cancer chemotherapy, reducing the drug resistance of the tumor cells can significantly improve the efficacy of chemotherapy. Fu LW demonstrated that TET was a highly effective multidrug resistance modulator *in vitro* and *in vivo*, without appearing to increase the toxicity of accompanying chemotherapy drugs (Fu et al., 2002). 2) “Characterization of tetrandrine, a potent inhibitor of P-glycoprotein-mediated multidrug resistance”: Fu LW demonstrated the multidrug resistance modulator activity *via* the KBv200 cells (Fu et al., 2004). Fu LW has made significant contributions to research on TET’s drug resistance reversal effect in tumor cells, bringing TET’s undiscovered pharmacological action within the researchers’ vision. 3) “Tetrandrine Inhibits Wnt/beta-Catenin Signaling and Suppresses Tumor Growth of Human Colorectal Cancer”: the activation of Wnt/beta-catenin pathway can lead to colon cancer, Wnt/beta-catenin pathway may be a promising target for colon cancer treatment. He BC observed that TET therapy can reduce the amount of beta-catenin protein in xenograft tumors (He et al., 2011), it’s a glad tiding for patients with colon cancer. Most of the top 10 cited articles were related to cancer therapy, indicating that cancer therapy is a hot topic in TET research field.

**TABLE 7:** Top 10 cited literatures.

Rank	Authors	Citations	Article title	Journal abbreviation	Date	Volume	Doi	Pubmed id
1	Fu LW	160	The multidrug resistance of tumour cells was reversed by tetrandrine *in vitro* and in xenografts derived from human breast adenocarcinorna MCF-7/adr cells	Eur J. cancer	2002	38	10.1016/S0959-8,049 (01)00356-2	11818209
2	Fu LW	146	Characterization of tetrandrine, a potent inhibitor of P-glycoprotein-mediated multidrug resistance	Cancer chemoth pharm	2004	53	10.1007/s00280-003-0742-5	14666379
3	He BC	128	Tetrandrine Inhibits Wnt/beta-catenin signaling and suppresses tumor growth of human colorectal cancer	Mol pharmacol	2011	79	10.1124/mol.110.068668	20978119
4	Pang ZQ	116	Lactoferrin-conjugated biodegradable polymersome holding doxorubicin and tetrandrine for chemotherapy of glioma rats	Mol pharmaceut	2010	7	10.1021/mp100277h	20957995
5	Kim DE	114	Natural Bis-Benzylisoquinoline Alkaloids-Tetrandrine, Fangchinoline, and Cepharanthine, Inhibit Human Coronavirus OC43 Infection of MRC-5 Human lung cells	Biomolecules	2019	9	10.3390/biom9110696	31690059
6	Liu CY	111	Tetrandrine induces apoptosis by activating reactive oxygen species and repressing Akt activity in human hepatocellular carcinoma	Int J. Cancer	2011	129	10.1002/ijc.25817	21128229
7	Bhagya N	109	Tetrandrine—A molecule of wide bioactivity	Phytochemistry	2016	125	10.1016/j.phytochem. 2016.02.005	26899361
8	Gong K	103	Autophagy-related Gene 7 (ATG7) and Reactive Oxygen Species/extracellular signal-regulated kinase regulate Tetrandrine-induced autophagy in human hepatocellular carcinoma	J. Biol Chem	2012	287	10.1074/jbc.M112.370585	22927446
9	Wang G	102	Herbal alkaloid tetrandrine: from an ion channel blocker to inhibitor of tumor proliferation	Trends Pharmacol Sci	2004	25	10.1016/j.tips. 2004.01.009	15058281
10	Meng LH	101	Tetrandrine induces early G (1) arrest in human colon carcinoma cells by down-regulating the activity and inducing the degradation of G (1)-S-specific cyclin-dependent kinases and by inducing p53 and p21(Cip1)	Cancer Res	2004	64	10.1158/0008-5472.CAN-04-0313	15604277

### 3.8 Most Influential Institutions

**FIGURE 9:**
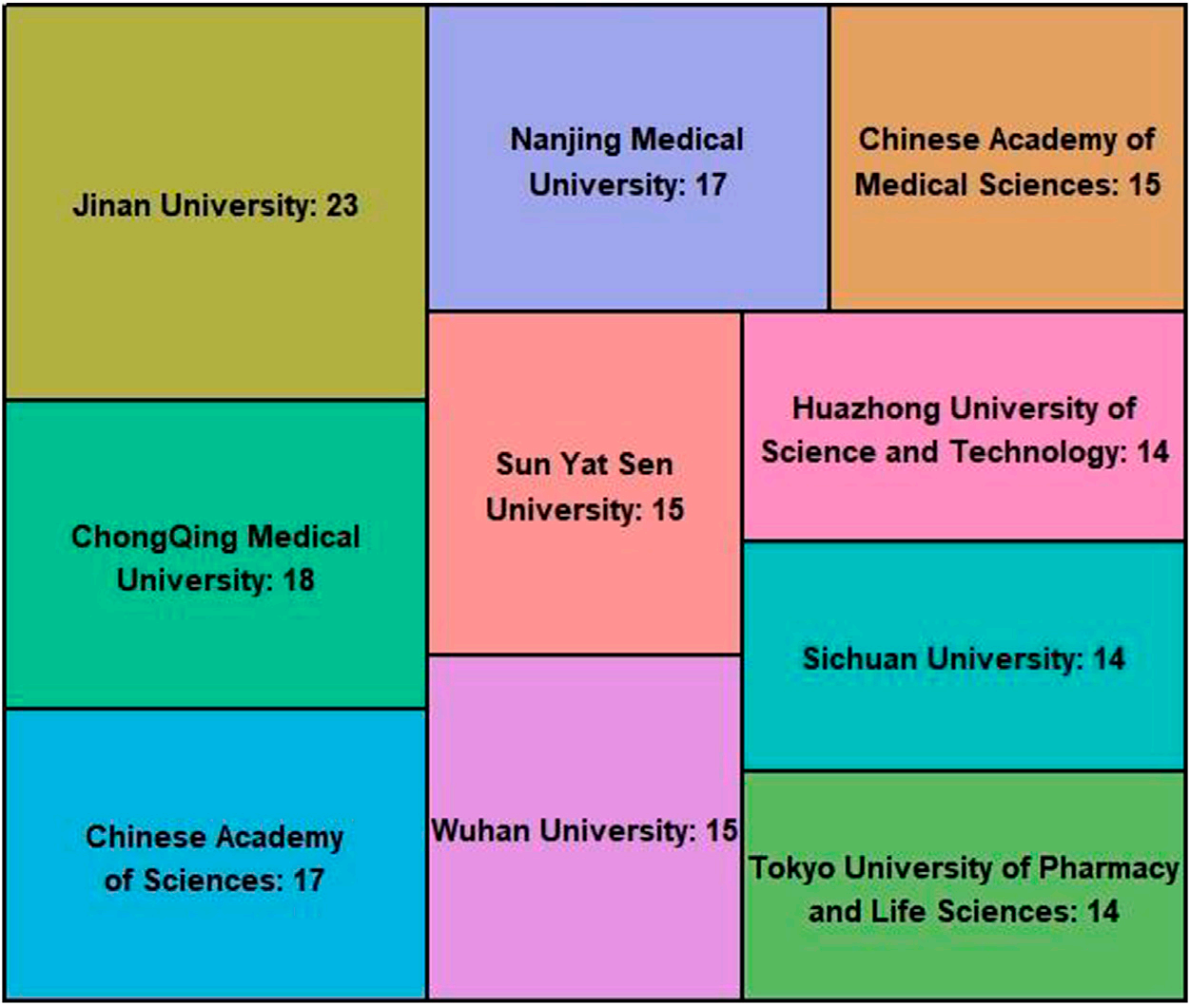
The top 10 most productive institutions. The data in the figure refer to the publication of the specific institution. The size of each board area is proportional to the number of articles.

**FIGURE 10:**
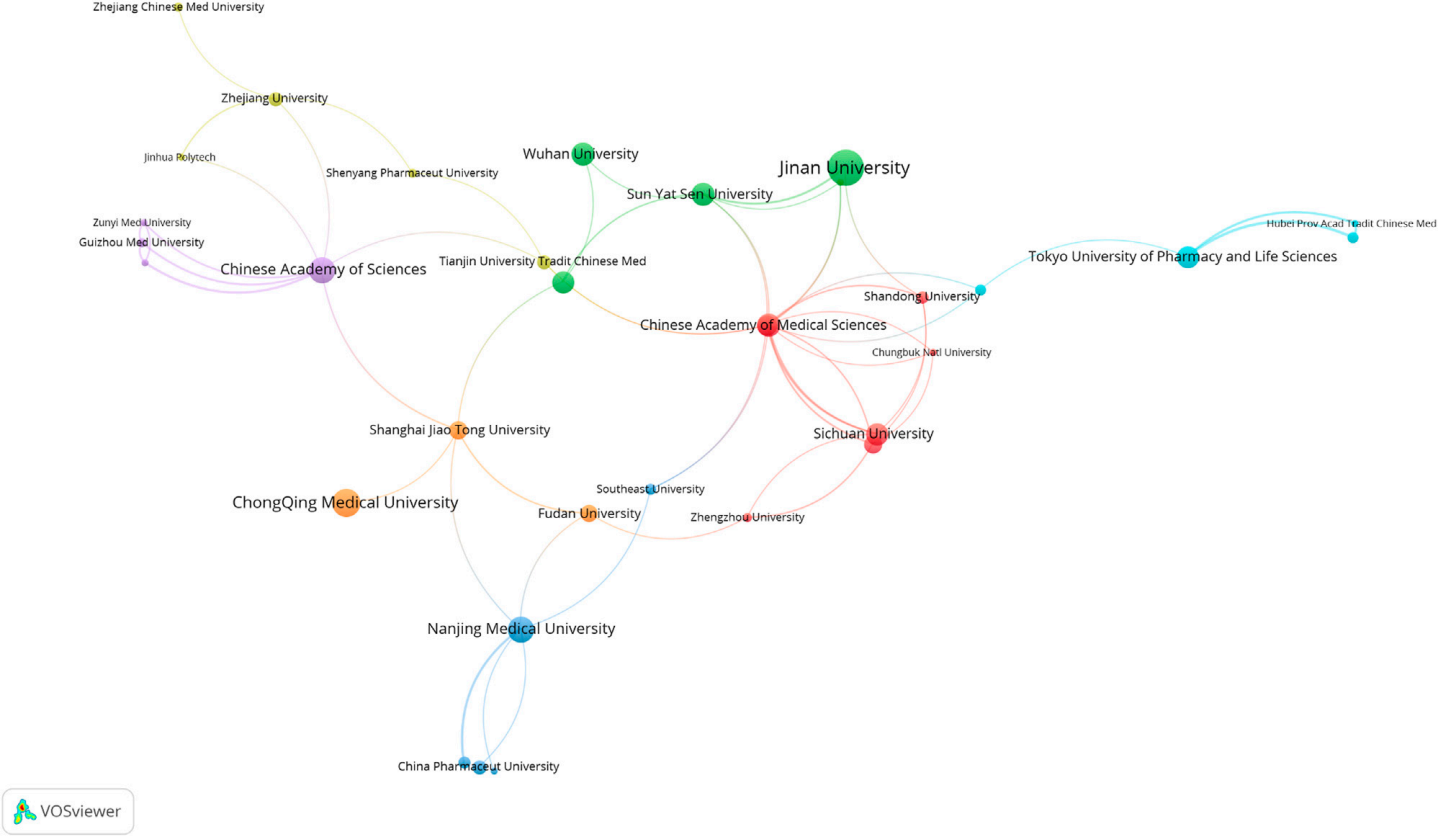
Network visualization of institutions collaboration.

The most productive institutions were represented in Figure 9, most of the influential institutions were from China. The top 10 most productive institution were: Jinan University, ChongQing Medical University, Chinese Academy of Sciences, Nanjing Medical University, Chinese Academy of Medical Sciences, Sun Yat Sen University, Wuhan University, Huazhong University of Science and Technology, Sichuan University, Tokyo University of Pharmacy and Life Sciences. Although Jinan University has the largest number of published articles, Jinan University has little cooperation with other institutions. However, Chinese Academy of Sciences has more cooperation work with other institutions. As shown in Figure 10, Chinese Academy of Sciences is an important collaborating center in the research field of TET.

## 4 Discussion

As a comprehensive method for analyzing the trend of literature publication, bibliometrics can provide scientific and insightful information for researchers to conduct their research work efficiently. At present, no bibliometric study in this field has been published. This study analyzed 482 articles from TET research field in recent two decades. Notably, the number of published articles in TET research field was in a relative low volume over the years. However, the number of articles published in 2016 was 2.44 times than that of 2008, the upward publication trend from 2001–2021 indicated that TET research is gaining traction. Increasing founding opportunities and more research investment seems to be the main factors contribute to the increasing prevalence of TET research. A downward trend from 2000 to 2021 in average citations showed that the latest articles has not been noticed by TET focused researchers. Notably, the level of average citations does not entirely represent the article’s quality because citations for previous publications are often higher than those for current items, citation lag contribute to this phenomenon.

The majority of the top 10 most productive authors were from China, and only three authors were from other countries, reflecting that China is predominant player in this research field. Zhang H. (Jinan Univ, Affiliated Hosp 1) was the most prolific author, Hirano T., Wang J. and Zhang Y. ranked next with 14 articles. Obviously, Zhang Y. and Zhang H. were both from Jinan Univ, indicating that Jinan Univ was significant contributor in this field. In order to illustrate the most prominent authors within this field in less bias, both the author’s publication ranking and citations ranking were included into the judging criterion. Despite the facts that Zhang Y. was the most prolific author, she was just a marginal contributor to the most of his studies. With a total of 11 articles (Rank 5) and 353 total citations (Rank 4), Cheng Y. seemed to be more influential in this research field. Among the top 10 authors distributed by total citations, most of their first paper was published in 2002. FU LW’s study in 2002 (Fu et al., 2002), which obtained the highest citation amount per paper in this research field, may provide guidance to TET focused researchers.

Most of the articles were came from China, only the United States, South Korea, and Japan have published more than 10 articles in this research field. The explanation to the dominant position of China maybe tetrandrine is frequently used in traditional Chinese medicine, therefore, Chinese researches have paid more attention for TET study than other countries. Other countries may be unable to conduct corresponding research independently due to the lack of medicinal material TET.

Acta Pharmacol Sin seemed to be the most influential journal in TET research field with both the largest number of publications and citations. However, none of the Top 10 highly cited articles was from Acta Pharmacol Sin. Cancer Chemoth Pharm was the second popular journal with a total of 280 citations, however, the 2020 JCR indicated that Cancer Chemoth Pharm was not an advanced journal. As the Rank 3 in the top 10 most cited journals, Int J. Oncol obtained a total of 272 citations and published 8 articles. Int J. Oncol published more article than Acta Pharmacologica Sinica during 2015–2020 revealed that the decreased predominance of Acta Pharmacologica Sinica in TET field. With 6.15 IF, Acta Pharmacologica’s high standard for included literature was also the main cause for the decrease of publication. All in all, after considering 2020 JCR, publications and citations, Acta Pharmacologica was still the authoritative journal in this field.

## 5 Hotspots and Frontiers

Based on a neutral conjunction of top keywords and literature, we ascribe the research hotspots as follows: 1) TET for cancer treatment: 256 items were retrieved *via* the search term (Title = Tetrandrine And Topic = apoptosis), the majority of articles related to the keyword “apoptosis” focused on exploring TET’s anti-cancer properties, TET has been demonstrated to suppress tumor tissue through a variety of mechanism. literatures with high citation times were selected out for further hotspots illustration. In Liu CY’s study, he observed that TET can activate reactive oxygen species and contribute to human hepatocellular carcinoma apoptosis (Liu et al., 2011), similarly, Wan J. demonstrated that Reactive oxygen species (ROS)/Akt signaling mediates the synergistic antitumor efficacy of sorafenib in conjunction with tetrandrine (Wan et al., 2013). Other useful action mechanism such as Wnt/beta-Catenin Signaling inhibition effect (He et al., 2011), ion channel blocker activity (Wang et al., 2004), drug resistance reversal effect (Zhu et al., 2005) are also of great significance. 2) Pharmacokinetics exploration in TET: the top 10 most frequent keywords “Pharmacokinetics” and keywords in the green cluster (Figure 6) indicated that Pharmacokinetics exploration in TET is another hotspot. *Via* the rats model, Li Z investigated the pharmacokinetics of fangchinoline and tetrandrine following single drug treatment and mixing with other effective ingredient in Chinese traditional prescription (Li et al., 2009). Liu CX invented a self-nanoemulsifying drug delivery system for enhancing bioavailability physicochemical of TET (Liu et al., 2018). Li JJ compared the effect of different chitosan lipid nanoparticles on improving ophthalmic TET delivery (Li et al., 2020). These studies provide fertile soil for TET further application in clinical practice. 3)TET for arthritis treatment: TET has been found to have an anti-arthritis impact in a great number of latest research, its anti-arthritis mechanism is visible in a variety of pathway. Liu QY demonstrated that TET reduces neutrophil activity in mice rheumatoid arthritis model (Lu et al., 2022). Zhong ZY discovered that TET protects 0variectomized mice from bone Loss by inhibiting RANKL-Induced Osteoclastogenesis. TET was also reported to play a critical role in improving rheumatoid arthritis prognosis through inhibiting pro-inflammatory factors *via* NF-kappa activation (Gao et al., 2016) and down-regulating the expressions of Rac1, Cdc42, RhoA GTPases and activation of PI3K/Akt and JNK signaling pathways (Lv et al., 2015).

## 6 Strengths and Limitations

Strengths of this study include visualization of author and institutional collaborations, prediction of research hotspots, and visualization of journal publications according to the temporal dimension. The full use of WOS database is also an advantage of this study. Compared with Pubmed, the documents data exported from the WOS database is more complete and show more convenience to our investigation. There are a group of limitations to this study. First, VOSviewer cannot visualize keywords and relevant time data in the same graph, resulting in subdiscipline classifications that are out of sync with time, neglect of time data may also lead to the hotspot prediction bias. Second, inclusion of other databases like PubMed and Scopus may provide a higher volume of published documents and comprehensive results. Although confining the search scope to title can improve the accuracy of our search results, it is inevitable that articles irrelevant to our topic may be included. Last, we might have neglected several studies on TET research if the authors did not put our study inclusion characteristics in the article titles.

## 7 Conclusion

The number of articles in tetrandrine research field increased year by year. The most influential author is Cheng Y. (Natl Taiwan Univ Hosp). China was the largest contributor to tetrandrine research, followed by the United States. Acta Pharmacologica Sinica remained the main publication related to tetrandrine research. Chinese Academy of Sciences, is expected to be a good candidate for collaborative research in this field. The use of tetrandrine in cancer treatment, could be the research subject areas to follow in years to come.

## Data Availability

The original contributions presented in the study are included in the article/supplementary material, further inquiries can be directed to the corresponding authors.
